# Toll-like Receptor Ligands Enhance Vaccine Efficacy against a Virulent Newcastle Disease Virus Challenge in Chickens

**DOI:** 10.3390/pathogens12101230

**Published:** 2023-10-11

**Authors:** Chang-Won Lee, Abhijeet Bakre, Timothy L. Olivier, Sonsiray Alvarez-Narvaez, Telvin L. Harrell, Steven J. Conrad

**Affiliations:** 1 Exotic and Emerging Avian Viral Diseases Research Unit, Southeast Poultry Research Laboratory, U.S. National Poultry Research Center, Agricultural Research Service, U.S. Department of Agriculture, Athens, GA 30605, USA; abhijeet.bakre@usda.gov (A.B.); tim.olivier@usda.gov (T.L.O.); 2 Endemic Poultry Viral Diseases Research Unit, Southeast Poultry Research Laboratory, U.S. National Poultry Research Center, Agricultural Research Service, U.S. Department of Agriculture, Athens, GA 30605, USA; sonsiray.alvareznarvaez@usda.gov (S.A.-N.); telvin.harrell@usda.gov (T.L.H.); steven.conrad@usda.gov (S.J.C.)

**Keywords:** Newcastle disease, toll-like receptors, imiquimod, ODN-1826, interferons, mucosal vaccine

## Abstract

To enhance the efficacy of the current Newcastle disease vaccine, we have selected potential adjuvants that target well-characterized pattern recognition receptors: the toll-like receptors (TLRs). Imiquimod is a small-molecule activator of TLR7, which is a sensor of dsDNA. ODN-1826 is a mimetic of CpG DNA and ligates TLR21 (a chicken homologue of TLR9 in mammals). The activation of TLRs leads to antiviral responses, including the induction of type I interferons (IFNs). In this study, birds were vaccinated intranasally with a live LaSota strain with or without imiquimod or ODN-1826 (50 µg/bird). Two weeks after vaccination, the birds were challenged with a virulent Newcastle disease virus (chicken/CA/212676/2002). Both adjuvants (imiquimod or ODN-1826) induced higher and more uniform antibody titers among vaccinated birds compared with the live vaccine-alone group. In addition, adjuvanted vaccines demonstrated greater protective efficacy in terms of the reduction in virus-shedding titer and the number of birds shedding the challenge virus at 2 and 4 days post-challenge. A differential expression of antiviral and immune-related genes was observed among groups from tissues (Harderian gland, trachea, cecal tonsil, and spleen) collected 1 and 3 days after treatment. These results demonstrate the potential of TLR-targeted adjuvants as mucosal vaccine enhancers and warrant a further characterization of immune correlates and optimization for efficacy.

## 1. Introduction

Newcastle disease (ND) is one of the most important poultry diseases worldwide and one of the notifiable animal diseases by the World Organization for Animal Health (WOAH, previously known as OIE) [1]. ND is a highly infectious disease caused by virulent avian orthoavulavirus 1 (AOAV-1), which is commonly known as avian paramyxovirus 1 (APMV-1) or Newcastle disease virus (NDV) [2]. In spite of routine immunization with live and killed vaccines incorporated into vaccination programs, ND and low-virulent AOAV-1-related problems remain a considerable cause of economic loss to the poultry industry [2,3]. Therefore, complementary strategies to enhance vaccine efficacy via boosting protective host immune responses are continuously being investigated. Although AOAV-1 has a single serotype, the large amount of genetic variation among isolates leads to the classification of AOAV-1 into two classes (class I and II), and there are many genotypes within class II viruses [4]. The mismatch between the field and vaccine strains has been considered one of the possible reasons for vaccination failure [5,6].

All pathogens encode specific molecular signatures known as pathogen-associated molecular patterns (PAMPs); PAMPs are recognized by pathogen recognition receptors (PRRs) in/on the host cell. Toll-like receptors (TLRs) constitute one class of PRRs involved in the recognition of viral and bacterial PAMPs [7,8]. The ligation of a PAMP triggers a cascade of intracellular activity that results in the upregulation of genes responsible for innate immunity and antiviral activity [7]. For this reason, several TLR ligands have been exploited as candidates for innate immune response enhancers in hosts against both bacterial and viral pathogens [9,10]. 

Chicken TLR21 is a functional homologue of mammalian TLR9 and recognizes synthetic ligands such as CpG-oligodeoxynucleotides (CpG-ODNs) [11,12]. It was shown that chicken and duck TLR21s, compared with mammalian TLR9 and fish TLR21, have a broad CpG-ODN sequence recognition profile, which may be advantageous in selecting and optimizing CpG-ODNs for poultry use [13]. After the first demonstration of the stimulatory effect of CpG sequences on the humoral response in chickens [14], different CpG-ODNs have been evaluated for prophylactic use and as vaccine adjuvants against different poultry pathogens [15,16,17,18,19,20,21,22]. CpG-ODNs are classified based on their structural and immunomodulatory attributes (e.g., class A, B, and C). In chickens, most studies have been conducted with class B CpGs, which are monomeric and known to promote plasmacytoid dendritic cell maturation and stimulate B cells [23]. 

Imiquimod (R837) is a small-molecule guanosine analog and an activator of TLR7 that has been extensively tested in mice and humans [24]. In chickens, an intraperitoneal injection of imiquimod upregulated IFN-γ, IL-1β, and IL-6 gene expression in the spleen [25], and in splenocytes isolated from duck and geese, imiquimod induced IFN-α, IL-1β, and IL-6 gene expression [26,27]. Imiquimod has also been tested as one of the many adjuvant components for enhancing inactivated vaccines against avian influenza in chickens and ducks, but its individual role in enhancing vaccine efficacy is unclear [28,29]. Resiquimod (R848), a structurally related immunomodulator, has been more frequently tested in chickens (compared with imiquimod) [30,31,32,33,34] and tested as an adjuvant for an inactivated NDV vaccine, where an enhanced humoral and cellular immune response was observed [35,36].

In this study, we selected two TLR ligands, ODN-1826 and imiquimod, to test their potential as adjuvants for a live NDV vaccine. ODN-1826 is a class B CpG ODN and was shown to be the most efficacious in reducing oral virus shedding against a low pathogenic avian influenza virus challenge compared with other class B or different classes (A and C) of CpG ODNs [18]. Imiquimod has not been tested in chickens for prophylactic use or as a sole vaccine adjuvant without being combined with other adjuvants, although extensive mammalian studies have shown its immunostimulatory effect [24]. In addition, in a study by Tachibana et al. [25], imiquimod treatment did not induce any negative behavioral and physiological changes, while resiquimod induced anorexia, hypoactivity, hypothermia, an inhibition of crop emptying, and a stress response. With a focus on enhancing mucosal immunity at the initial site of infection to provide better protection, we treated the birds intranasally with different combinations of a live NDV vaccine and ODN-1826 or imiquimod. The potential adjuvant effect of ODN-1826 and imiquimod is described in relation to enhancing antibody responses, reducing challenge virus shedding, and inducing antiviral genes in different tissues. 

## 2. Materials and Methods

**Eggs and chickens.** Eggs derived from the specific pathogen-free (SPF) white Leghorn flock maintained at the U.S. National Poultry Research Center (USNPRC, Athens, GA, USA) were used for virus propagation and back titrations as well as hatching the birds for animal experiments. The chickens were randomly placed into treatment groups, identified with a wing band, and housed in negative pressure isolation units in BSL-2 facilities during treatment or the vaccination period. The birds were moved to an enhanced BSL-3 facility at the time of challenge. Birds had ad libitum access to food and water throughout the experiment. The USNPRC Institutional Animal Care and Use Committee approved all animal experiments.

**Viruses and TLR ligands.** Working stocks of both vaccine (LaSota) and challenge (California/212676/2002 (CA02)) viruses were obtained from the Southeast Poultry Research Laboratory repository and propagated in 9–11-day-old eggs [37]. LaSota is a class II, genotype II virus, while CA02 is a virulent viscerotropic virus that belongs to class II, genotype V lineage [4]. ODN-1826 (InvivoGen, San Diego, CA, USA, Cat. # vac-1826-1) and imiquimod (InvivoGen, Cat. # vac-imq) were diluted in nuclease-free water to the manufacturer’s suggested concentration to create stock solutions.

**Experimental design.** SPF birds (16 birds/group) were either mock treated, treated with adjuvant or vaccinated at 14 days of age with or without adjuvant (Table 1). The LaSota vaccine (10^6^ EID_50_/0.2 mL) was either mixed with PBS (Mock) or adjuvant (50 µg/bird) and administered into the eye (0.1 mL) and choanal cleft (0.1 mL). 

At 1 and 3 days post-vaccination, respectively, 3 birds per group were euthanized and tissues (Harderian gland, trachea, cecal tonsil, and spleen) were collected into screw-cap vials containing 500 μL of cold TRIzol reagent (Ambion, Carlsbad, CA, USA, Cat. # 10296028) and placed in ice. Samples were kept at −80 °C until being processed for RNA extraction. Two weeks after vaccination, blood samples were collected to determine the anti-NDV antibody titer in the sera, and 10 birds per group were challenged with virulent NDV (CA02, 10^5^ EID_50_/0.1 mL) by administering the virus into the eye (0.05 mL) and choanal cleft (0.05 mL). Back titration of the infectious challenge virus after dilution [38] confirmed the challenge dose to be 10^5.1^ EID_50_/0.1 mL.

At 2 and 4 days post-challenge, oropharyngeal and cloacal swabs were collected to analyze the relative viral RNA amount by real-time RT-PCR as described below. Samples were collected using a dry swab (Puritan Medical Products Company LLC, Guilford, ME Cat. #: 89194-894). All swabs were placed in 1.5 mL brain–heart infusion (BHI) medium with penicillin (2000 units/mL; Sigma Aldrich, St. Louis, MO, USA), gentamicin (200 μg/mL, Sigma Aldrich) and amphotericin B (5 μg/mL, Sigma Aldrich) and stored at −80 °C until use. 

**Virus titration and hemagglutination inhibition (HI) antibody test.** The vaccine and challenge viruses were titrated in 9-11-day-old eggs using the Spearman–Karber method [38]. HI antibody tests were conducted on both pre-challenge and post-challenge sera in 96-well microtiter plates against the vaccine (LaSota strain) and challenge (CA02 strain) viruses as described [1].

**RNA extraction and real-time RT-PCR.** RNA was extracted from each swab sample using the MagMAX 96 AI/ND viral RNA isolation kit (Thermo Fisher Scientific, Vilnius, Lithuania, Cat. #: AM1835) with a KingFisher^TM^ Purification System (Thermo Fisher Scientific, Finland, Cat. #: 9016016) following the manufacturer’s instructions. The extracted RNA was used to quantify the challenge virus RNA by a real- time RT-PCR using AgPath-ID One-Step RT-PCR Reagents (Life Technologies, Austin, TX, USA, Cat. #: 4387391) and primers and the probe specifically targeting the fusion gene of virulent NDVs [39].

The RNA from tissue samples was extracted as described below for both transcription analysis and detection of the vaccine (LaSota strain) virus. Real-time RT-PCR was conducted with 200 ng of total RNA extracted from each tissue using primers and probe targeting polymerase gene (L) of AOAV-1 viruses using previously published conditions [40]. 

**Extraction of RNA from tissue and conversion to cDNA.** Each tissue sample in 500 μL TRIzol reagent was homogenized by hand using a plastic pestle (Avantor, Suwanee, GA, USA, Cat. #: KT749521-1500). Then, 50 μL BCP Reagent (1-Bromo-3-Chloropropane, Molecular Research Center, Inc, Cincinnati, OH, USA, Cat. #: BP151) was added to homogenates, and the tubes were centrifuged at 12,000× *g* at 4 °C for 15 min in an Eppendorf 5804R tabletop centrifuge. The upper, colorless layer was removed to a clean tube, and the RNA from this layer was precipitated with 0.5 mL 2-propanol (Fisher Scientific, Waltham, MA, Cat. #: A516-500). Then, the tubes were centrifuged at 12,000× *g* at 4 °C for 10 min. The liquid phase was removed, and the pellet was washed once with 75% ethanol before being centrifuged at 12,000× *g* at 4 °C for 5 min. The precipitated RNA was air-dried and resuspended in 50 μL nuclease-free water (Ambion, Austin, TX, USA, Cat. #: AM9937). RNA concentrations were determined by spectrophotometry using a NanoDrop 1000 spectrophotometer (Thermo Scientific, Monroe, GA, USA, Cat. #: ND-1000). RNA was converted to cDNA using the LunaScript RT SuperMix Kit (New England BioLabs, Ipswich, MA, USA, Cat. #: E3010L) using 500 ng total RNA in a 20 μL reaction as per manufacturer’s recommended conditions. The 20 μL cDNA reaction was diluted 20-fold in a total of 400 μL nuclease-free water.

**Transcription analysis.** For the quantitative gene expression analysis, primers were newly designed for each target gene using the Integrated DNA Technologies PrimerQuest software tool (https://www.idtdna.com/Primerquest/Home/Index, accessed on 17 July 2023), and the NCBI transcript reference numbers for each gene as provided in Table 2. All primers except for MX1 and IFNω1 (which are intronless or single-exon genes) spanned an exon–intron overlap junction (Table 2). 

**Figure 1:**
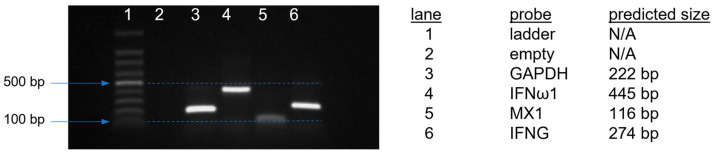
Validation of specificity of primer pairs. Each primer pair was tested to ensure it produced a unique amplicon at the predicted mass. The amplicons resulting from 40 cycles of amplification were run on a 2% agarose gel at 90 V for 60 min. All bands are unique and match their predicted mass.

Primers were designed using a requirement for optimal binding at 63 °C, while all other parameters were set to default. Each primer pair was checked for the specificity, uniqueness of the product amplicon, and correctness of size of the amplicon product before use by agarose gel (Figure 1) in which 10 μL of qPCR product from an amplification performed on material derived from Harderian gland in this experiment, under the conditions described (above), was run on a 2% agarose gel (UltraPure agarose, Invitrogen, Carlsbad, CA, USA, Cat. #16500-500). In addition, each qPCR product exhibited a single, sharp melting point, which was consistent from run to run.

Each 25 μL quantitative PCR (qPCR) reaction contained 12.5 μL of 2X Luna Universal SYBR qPCR Master Mix (New England BioLabs, Cat. #: M3003E), 10 μL cDNA template (prepared as above), 1.25 μL of 4 μM forward/reverse primer solution, and 1.25 μL nuclease-free water. After exposure for 3 min to 95 °C, each cycle of elongation was completed by 15 s exposure to 95 °C followed by 60 s exposure to 63 °C. Quantitative PCR runs were completed on an Applied Biosystems 7500 real-time PCR system (Applied Biosystems, Foster City, CA, USA, Cat. #: 4351106) for 40 cycles followed by a melting curve from 63 to 95 °C in 0.5 °C increments.

For each condition, three technical replicates were completed for each of the three target genes of interest (IFNω1, MX1, and IFNγ). Differential gene expressions were normalized using chicken glyceraldehyde-3-phosphate dehydrogenase (GAPDH) gene expression. Relative mRNA expression levels were determined as fold changes over the mock group as previously described using the 2^−*ΔΔCT*^ method [41,42].

**Statistical analysis.** Statistical analysis was performed using GraphPad Prism, version 9.5.1 (GraphPad Software LLC, San Diego, CA, USA). A one-way analysis of variance (ANOVA) followed by Tukey’s post hoc test was used for comparing the antibody response, viral RNA titer, and gene expression level in transcription analysis.

## 3. Results

### 3.1. Hemagglutination Inhibition (HI) Antibody Titer at 2 Week Post-Vaccination 

**Figure 2:**
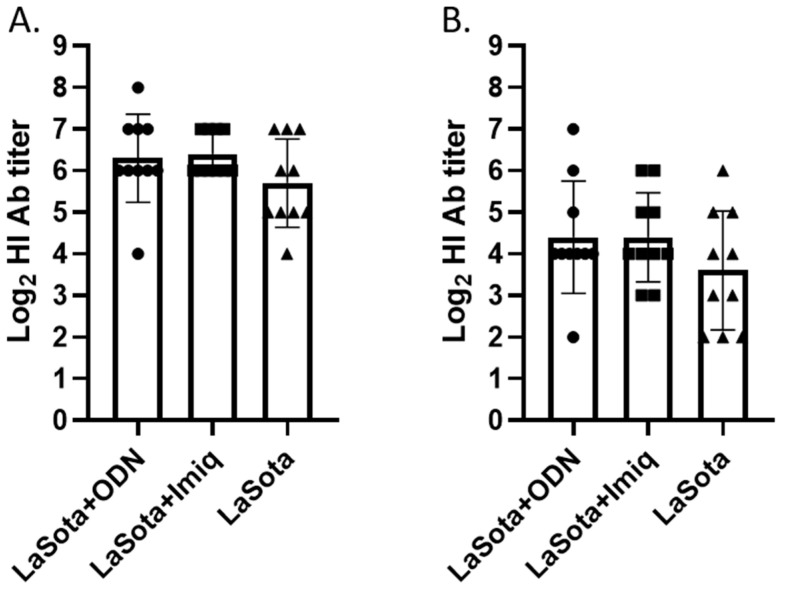
Post-vaccination HI antibody titer against LaSota (**A**) and CA/02 (**B**) antigens. Interleaved bars indicate mean titer with standard deviation. ODN = ODN-1826; Imiq = imiquimod.

HI antibody titer was measured using both vaccine (LaSota) and challenge (CA/02) viruses as antigens. Although the overall HI antibody titer was about 4-fold higher when LaSota antigen was used for the assay compared with the CA/02 antigen, a similar trend of antibody titer difference was observed among the vaccine groups. Higher mean and median titers in groups vaccinated with adjuvants compared with the vaccine-alone group were observed using both LaSota and CA/02 antigens, although the difference was not statistically significant (Figure 2). 

### 3.2. Morbidity and Mortality Post-Challenge 

All birds in unvaccinated groups (PBS control and adjuvant-alone groups) showed typical clinical signs of virulent NDV challenge which include severe depression, conjunctivitis, periocular edema, ruffled feathers, and open-mouthed breathing, and they either died or were euthanized between 2 and 4 days post-challenge. All birds in LaSota-alone or adjuvanted-vaccinated groups remained healthy throughout the experiment. The dead or euthanized birds were excluded from the swab sample collection to avoid variation in sample quality.

### 3.3. Viral RNA Detection from Oropharyneal and Cloacal Swabs 

**Figure 3:**
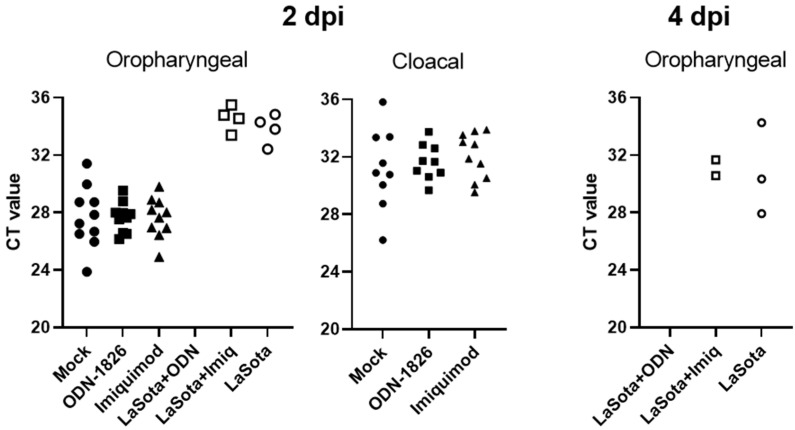
Oropharyngeal and cloacal virus RNA detection indicated as cycle threshold (CT) value determined by real-time RT-PCR. Data for unvaccinated groups (mock, ODN-1826, and imiquimod) at 4 dpi are not shown because all birds were either dead or euthanized. RT-PCR negative samples (CT values greater than 36) are also not shown in the figure. dpi = days post-infection; ODN = ODN-1826; Imiq = imiquimod.

Adjuvant treatment alone did not reduce challenge virus shedding, which was determined by measuring the amount of viral RNA (which is inversely correlated with the cycle threshold (CT) value in Figure 3) from oropharyngeal and cloacal swabs. At 2 days post-infection (dpi), all 10 birds (100%) in the unvaccinated groups (PBS, ODN-1826, or imiquimod treated groups) demonstrated a larger amount of virus from the oropharyngeal swab (lower CT values) compared with the birds in the three vaccinated groups, where no or only a few birds showed lower amount of virus (0% in ODN-1826-adjuvanted group and 40% in imiquimod-adjuvanted or LaSota-alone groups, respectively) (Figure 3). In cloacal swabs, only unvaccinated birds shed the virus (90% in mock or ODN-1826 groups and 100% in the imiquimod group, respectively) and no measurable viral RNA was detected from any of the vaccinated birds in the three groups. Shedding data at 4 dpi are not available for birds in unvaccinated groups since all birds were either dead or euthanized. Among the vaccinated groups, no viral RNA was detected from oropharyngeal swab in the ODN-1826-adjuvanted group. The LaSota-alone and imiquimod-adjuvanted groups showed similar amounts of virus shedding from 20% and 30% of the birds, respectively. Cloacal shedding of the challenge virus was only detected from one bird (CT value of 34.3) in the LaSota-alone vaccinated group at 4 dpi. 

### 3.4. HI Antibody Titer at 2 Week Post-Challenge 

**Figure 4:**
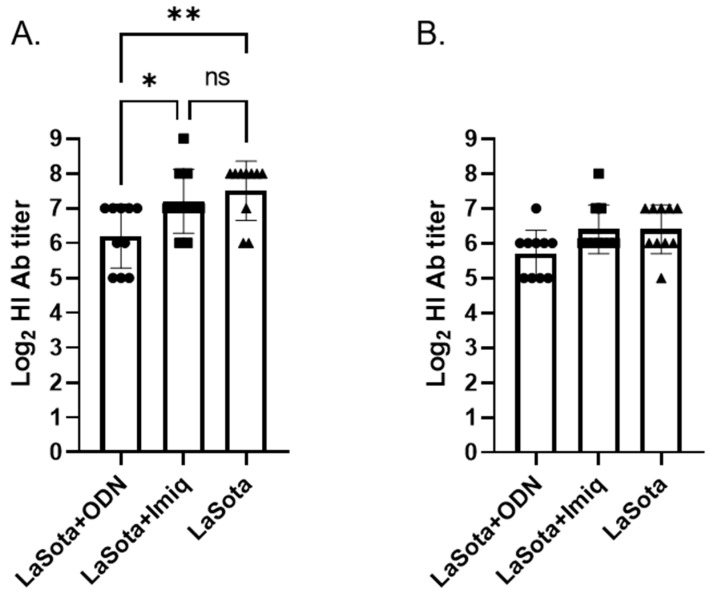
Post-challenge HI antibody titer against LaSota (**A**) and CA/02 (**B**) antigens. Interleaved bars indicate mean titer with standard deviation. * *p* < 0.05, ** *p* < 0.01, ns = non-significant. ODN = ODN-1826; Imiq = imiquimod.

Significantly different HI antibody titers (*p* = 0.0082 and *p* = 0.0457, respectively) among vaccinated groups were observed at 2 week post-challenge in one-way ANOVA analysis, although Tukey’s post hoc pairwise comparison could not find significant differences between vaccine groups when using CA/02 antigen (Figure 4B). Based on the HI test using LaSota antigen, a significant difference was observed between ODN-1826 and LaSota as well as between the ODN-1826 and imiquimod-adjuvanted vaccine groups (Figure 4A). Compared with the post-vaccination HI antibody titer (Figure 2), less than or approximately a 2-fold increase in titer was observed in the ODN-1826 and imiquimod-adjuvanted groups, respectively, while more than a 4-fold increase in titer was observed in the LaSota-alone group. The HI antibody data correlate with virus-shedding data (Figure 3) where a significant reduction in virus shedding was observed in the ODN-1826 group. 

### 3.5. IFN and IFN-Stimulated Gene Expression after Adjuvant Treatment or Adjuvanted Vaccination

**Figure 5:**
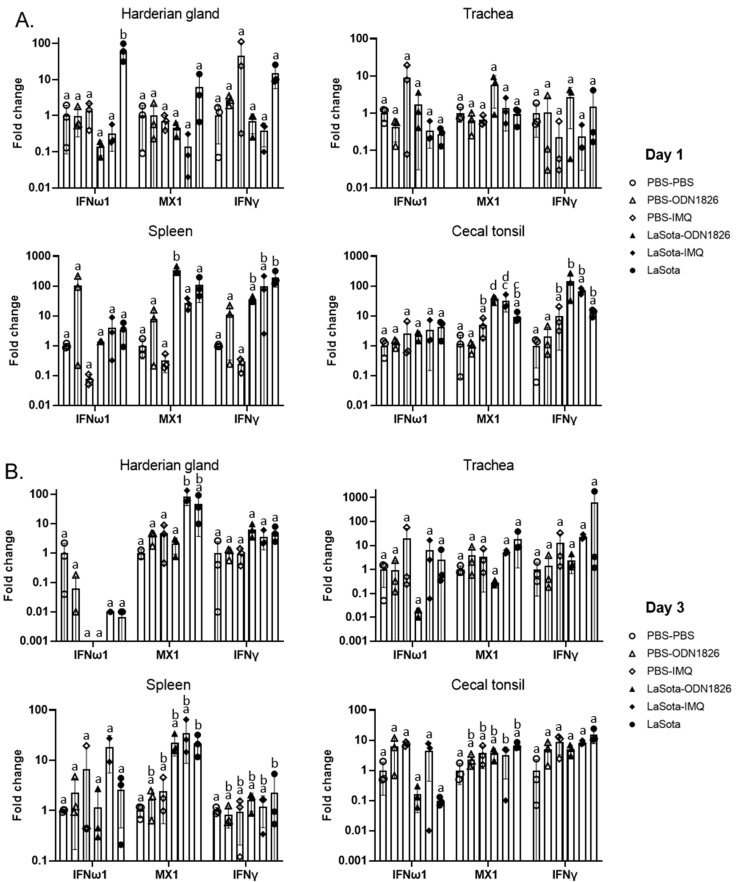
Effect of adjuvant and adjuvanted vaccine on antiviral gene expression in tissues at 1 day (**A**) and 3 days (**B**) after treatment. Fold change is normalized to housekeeping gene and plotted on a log10 scale, and downregulated gene expressions are plotted between 0 and 1. Negative values cannot be log transformed and are not shown. The values represent the average of triplicate samples from each group with standard deviation and are expressed as the relative mRNA level over the PBS-treated control group. Letters indicate statistical groups at a *p* value < 0.05.

At 1 day post-treatment, the effect of adjuvant alone in inducing antiviral gene expression was minimal in all tissues, and although the adjuvant-treated groups showed an upregulation trend (especially in spleen and cecal tonsil where the ODN-1826 group showed higher fold changes in all three genes in the spleen, and imiquimod showed a higher expression level in all three genes in the cecal tonsil compared with the PBS control group), the difference was not significant due to the high variation in expression levels among samples within the group (Figure 5A). The expression levels of all three genes in the spleen and cecal tonsils were higher in the vaccinated groups compared with the unvaccinated groups in general, and many of the differences were statistically significant. The LaSota-alone vaccinated group showed higher fold changes in all three genes in the Harderian gland compared with the two other adjuvanted vaccine groups, and the IFNω1 (alias IFNβ) expression level was significantly higher than in all the other five groups. Significant differences in the antiviral gene expression levels among the vaccinated groups were detected with the MX1 gene from the spleen, where the ODN-1826-treated group showed significant upregulation when compared with the LaSota-alone or the imiquimod-adjuvanted group, and also from the cecal tonsil, where the expression level in the ODN-1826 group was significantly higher than in the LaSota-alone group.

At 3 days post-treatment, a similar trend of higher fold change was observed in the adjuvant-treated groups compared with the PBS control group in both cecal tonsil and spleen for all three genes with the exception of IFNγ in the spleen (Figure 5B). As observed in the day 1 samples, the vaccine groups showed many significant increases in MX1 and/or IFNγ gene expression compared with the PBS control group in different tissues. However, the difference in gene expression level among vaccinated groups was not clear with the exception that MX1 expression was significantly higher in the Harderian gland with ODN-1826 adjuvantation compared with imiquimod adjuvantation. 

### 3.6. Vaccine Virus Detection from Tissues

Overall, a slightly higher amount of LaSota viral RNA was detected at 3 days post-vaccination compared with 1 dpi in the Harderian gland, spleen and cecal tonsil. The amount of viral RNA detected varied the most in tracheal samples. The Harderian gland showed a significantly higher amount of viral RNA at 3 dpi compared with other tissues with the exception of tracheal tissues from the LaSota-alone and imiquimod-adjuvanted groups. In all tissues, no significant difference in viral RNA amount was observed among the three vaccinated groups. 

## 4. Discussion

The ODN-1826 or imiquimod treatment alone did not provide any prophylactic benefit in this study, which was expected because the birds were challenged 2 weeks after the treatment and the prophylactic effect of TLR ligand has been shown to rapidly decrease after 2–3 days of treatment [15]. Although not tested, we expect that the local innate and mucosal immunity provided by the TLR ligand alone may not be enough to provide adequate protection against virulent NDV challenge even if birds are infected within 3 days of treatment. 

To directly assess the antiviral response upon the TLR ligand treatment, we collected four different tissues at two different time points to examine the local and systemic immunostimulatory effects and to cover both early and later gene expression. Overall, the antiviral gene response was minimal and resulted in nonsignificant differences between the groups, although the adjuvant-alone-treated groups showed an upregulation trend of different genes especially in the cecal tonsil and spleen at both time points tested. CpG ODN is known to induce a Th1-biased immune response with the upregulation of IFNγ in chickens [43], and imiquimod and resiquimod have been shown to result in more IFN-α induction than other cytokines in mammalian cells [24]; however, we did not notice those trends in our study. It is also difficult to compare the transcription results from different studies due to varying factors including the route and dose of ligand treatment, tissues and time points analyzed, antiviral genes targeted, and not to mention the exact class of TLR ligand used in the study. For example, in a similar study conducted by Barjesteh et al. [18], 2-week-old birds were treated with 10 µg of ODN-1826 intranasally, and a small but significant increase in IFNβ (5-fold) and IFITM3 (2.27-fold) genes in the trachea and a 2-fold increase in PKR in the cecal tonsil at 18 h post-treatment was observed. IFNγ was not tested in the study. Similar data for imiquimod or resiquimod describing antiviral gene response after intranasal treatment in chickens are not available. In a study by Sachan et al. [35], intramuscular injection of resiquimod (50 µg) induced significant IFNα, IFNβ, and IFNγ gene expression (more than 50-fold) in the spleen, which peaked at 24 h post-treatment. However, without a direct comparison of imiquimod and resiquimod and two different routes (intranasal and intramuscular) at the same time, it is difficult to extrapolate the immune correlates of one study to the other. 

Among vaccinated groups, we observed an increase in antibody response in CpG-ODN and imiquimod-adjuvanted groups consistent with previous studies [20,21,22]. However, the effect was minimal in our study compared with previous studies where the antibody response was measured after two vaccinations/treatments. Nevertheless, the significant reduction in virus shedding and lower post-challenge antibody response observed in our study further support the beneficial effect of TLR ligand adjuvantation especially with the ODN-1826. Zhang et al. [21] conducted a similar study with ODN-2007 and the LaSota vaccine, and despite the high dose of vaccine and booster vaccination applied, even the adjuvanted vaccine induced mortality. In our experimental model, we do not see clinical signs or mortality in a single dose of LaSota-vaccinated birds against virulent challenge virus that is of a different genotype from the vaccine strain. Thus, we use reduction in virus shedding as the main criterion, which provides a quantitative value to compare the efficacy of the vaccine. A study by Talebi and Arky-Rezai [20] also tested intranasal treatment of ODN-2007 in enhancing live ND vaccine (Clone 30) response using broiler chickens. However, in addition to using commercial birds with maternal antibodies, the study had a limited scope of evaluating the antibody response and white blood cell counts. Future studies are warranted with the animal challenge model that can better evaluate and compare the enhanced protective efficacy among different adjuvants by using a reduced vaccine dose and/or weaker live vaccine strains than the LaSota strain in which multiple criteria (e.g., mortality, reduction in virus shedding, clinical signs, microscopic lesion scoring, etc.) can be applied for comprehensive evaluation.

No similar studies were previously conducted with imiquimod for direct comparison. In a study by Sharma et al. [35,36], two-week-old SPF chickens were intramuscularly immunized twice with inactivated NDV vaccine in combination with 50 µg of resiquimod. Inactivated NDV vaccine with resiquimod induced a significantly higher humoral and cellular immune responses and better protection than a commercial oil emulsion vaccine in terms of protecting birds from mortality. However, it is unclear about the practical value of vaccinating the chickens twice with a two-week interval, and the study design may have favored the quicker effect of resiquimod-adjuvanted vaccine compared with slow-releasing oil emulsion vaccine. It is possible that although safer than resiquimod [25], the immunostimulatory effect of imiquimod may be less than resiquimod in chickens, and additional studies that directly compare the safety, immunostimulatory and protective effect of the two immunomodulators are needed. 

**Figure 6:**
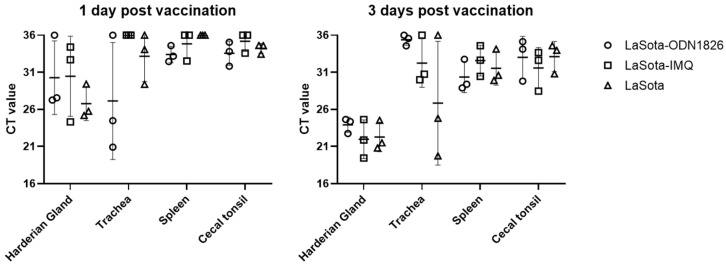
Vaccine viral RNA detection indicated as cycle threshold (CT) value determined by real-time RT-PCR. Samples where CT values were higher than the cut-off (36.0) were assigned the value of 36 for statistical analysis. Interleaved bars indicate mean value with standard deviation.

Live vaccination induced significant increases in gene expression compared with the PBS control group in different tissues. However, the difference in gene expression level among vaccinated groups was not clear other than the ODN-1826 treatment group showing a significant upregulation of the MX1 gene in the spleen and cecal tonsil at 1 day post-treatment and in the Harderian gland at 3 days post-treatment. Overall, the spleen and cecal tonsil demonstrated more differential gene expression than the Harderian gland or cecal tonsil, but we were not able to correlate the amount of vaccine virus detected in tissues with the treatment group and antiviral gene expression. The Harderian gland showed the highest amount of viral RNA at both time points tested, and no significant difference in viral RNA amount was observed among the three vaccinated groups in all tissues (Figure 6). 

Taken together, our study and previously published studies show that the kinetics of antiviral gene expression vary significantly by the tissues, type and potency of adjuvant used, and dose and routes of adjuvant application. It should also be noted that virus itself, whether as a vaccine or a challenge virus, will affect the antiviral gene response [18,42,44]. Recently, Liu et al. [45] utilized single-cell RNA sequencing of both host and viral transcriptomes in lung tissue from NDV-infected chickens, and the gene expression patterns and the IFN response in different putative trajectories were demonstrated. Considering the heterogeneity of cells in tissues and the different level of TLRs expressed by a variety of cell subsets, the comprehensive approach such as single-cell transcriptome analysis may pave the way for a better understanding and delineation of the immune correlates related to TLR ligands as prophylactic and vaccine adjuvant applications in future studies.

## Figures and Tables

**Table 1:** Experimental groups and treatments.

Group	Vaccine	Treatment Adjuvant
1. PBS control	none	none
2. Adjuvant only (ODN-1826)	none	ODN-1826
3. Adjuvant only (Imiquimod)	none	Imiquimod
4. Vaccine + Adjuvant (ODN-1826)	LaSota	ODN-1826
5. Vaccine + Adjuvant (Imiquimod)	LaSota	Imiquimod
6. Vaccine only	LaSota	none

**Table 2:** Primer sequences used in this study.

Primer Name	Primer Sequence	Reference ^1^
GAPDH_F5	5′-CTGAATGGGAAGCTTACTGGAATG-3′	NM_204305
GAPDH_R5	5′-CGCATCAAAGGTGGAGGAATG-3′	
IFNω1_F4	5′-ACAAGAAGCAAGCAGCCATC-3′	NM_001024836
IFNω1_R4	5′-GTGCGGTCAATCCAGTGTTT-3′	
MX1_F6	5′-CCAGACCTAGTGAACGAAGGAA-3′	XM_046901195.1
MX1_R6	5′-CAGAAGTCCATTTGCCCATAACA-3′	
IFNG_F1	5′-GTAGCTGACGGTGGACCTATT-3′	NM_205149
IFNG_R1	5’-AGAGTTCATTCGCGGCTTTG-3′	

^1^ NCBI transcript reference numbers.

## Data Availability

The raw data used for the analysis in this manuscript have been submitted to the National Agricultural Library under the identifier https://doi.org/10.15482/USDA.ADC/1529602.
